# An Observation of a Resident-as-Teacher Combined with Tutor Guided Hysteroscopy Teaching Program for Standardized Residency Training (SRT) in Obstetrics and Gynecology

**DOI:** 10.1155/2020/8855099

**Published:** 2020-07-30

**Authors:** Ye Yang, Li Yan Li, Lin Wei Sang, Bin Yong Yang, Ping Ya Zhu, Lei Dai, Wei Bao, Wei Liu, Fang Su Wu

**Affiliations:** ^1^Department of Obstetrics and Gynecology, Shanghai General Hospital, Shanghai Jiao Tong University School of Medicine, 85 Wujin Road, Hongkou, Shanghai 200080, China; ^2^Department of Orthopedics, Shanghai General Hospital, Shanghai Jiao Tong University School of Medicine, 85 Wujin Road, Hongkou, Shanghai 200080, China; ^3^Department of Education, Shanghai General Hospital, Shanghai Jiao Tong University School of Medicine, 85 Wujin Road, Hongkou, Shanghai 200080, China

## Abstract

**Objectives:**

The standardized residency training (SRT) program in China is an important link for continuing education and clinical work training for graduate students. The purpose of our study was to enable educators to maintain the effectiveness of hysteroscopy teaching techniques and make the standardized residency training students well experienced in surgery, thus demonstrating that higher efficiency of teaching can lead to better proficiency for surgery.

**Methods:**

We generated resident-as-teacher teaching round and tutor guided hysteroscopic surgery as well as a questionnaire based on the mastery degree of the basic theoretical knowledge and operational skills of hysteroscopy among seven junior residents and five senior residents of the Obstetrics and Gynecology Department, including four attending gynecologic surgeons of a hysteroscopy teaching program.

**Results:**

Senior residents felt confident to teach, while junior residents learned effectively through the teaching round. There were statistically significant differences in the whole operation time and the volume of distension fluid used between junior and senior residents (*p* < 0.05).

**Conclusions:**

This study acknowledges the need for new approaches to medical education for better characterization of the link between the use of teaching rounds through problem-based learning (PBL) discussion dominated by the residents themselves and overall surgical skills of teaching and learning.

## 1. Introduction

Medical graduates are not fully trained physicians when they leave medical school, so it is very important to build a connection between theoretical learning in college and practical training in the hospital. The standardized residency training (SRT) program in China is an important link for continuing education and receiving system-based clinical work training for students who have completed basic theoretical courses in a medical college [1]. It is defined as residents who participate in different departments to carry out systematic training for three years to gain overall improvement in professional competency including theoretical knowledge, clinical skills, teamwork competency, and communication with patients [2]. Residents are divided into Grade 1, Grade 2, and Grade 3 by the years they trained. Junior residents were defined as those in the Grade 1 to 2 SRT stage which correspond to the first to the second year of postgraduate training, while senior residents were defined as those in Grade 3 SRT stage in the third year of postgraduate training. Taking example for residents in the Obstetrics and Gynecology Department, they rotate between different departments including the gynecology ward and obstetrics ward each for 4 months in Grade 1, 6 months in Grade 2, and 3 months in Grade 3, and the outpatient department for 4 months in Grade 1, and the general surgery ward and pediatrics ward as well as medical imagery department each for 2 months in Grade 3.

The training process comprises standardized courses to facilitate the acquisition of the required skills. In Grade 1, basic skills will be trained, and in Grade 2 to 3, clinical skills and theoretical knowledge will be thoroughly studied and mastered. The residents should pass the theoretical and operating comprehensive examination before they graduate from the SRT program; then, they could become a specialist doctor. Currently, SRT is conducted in progress in most hospitals according to the Ministry of Health guidelines, while educators have long struggled with how best to act in teaching [3]. In China, patients have always been prejudiced against young doctors as residents lacked adequate experience. Once a surgical complication develops, it causes disputes with patients [2]. Therefore, senior doctors do not dare to allow residents to perform an operation independently, making surgical training even more difficult. Thus, a more effective and faster independent operation education is required for the training of residents.

Hysteroscopic surgery (HS) is an important subject taught in the SRT program during residents doing a clinical rotation in the gynecological ward. It is a vital technique for identifying many typical manifestations of gynecological diseases, such as uterine anatomy, normal and abnormal uterine morphologies, dysfunctional uterine bleeding, endometrial polyps, endometrial hyperplasia, submucosal uterine fibroids, endometrial cancer, mediastinal uterus, intrauterine adhesions, and foreign bodies in the uterine cavity [4]. However, compared with traditional open and laparoscopic surgeries, HS is a single-person operation that has a certain degree of surgical difficulty for beginners and requires a relatively long learning process [5]; therefore, it is a great challenge for teachers to physically show trainees how to accomplish hysteroscopic surgery rather than describe how to do it.

Different from the theoretical knowledge taught in medical school, teaching round (TR) was conducted in the hospital so that residents could learn real clinical cases. There are two stages of TR instructions: taught at the bedside and taught in the classroom. In bedside teaching, the trainees can observe behaviors of teachers with respect to how they handle introductions, address patients' concerns, elicit key details, and ask permission to examine and explain symptoms [6]. The second stage includes complex discussions on the etiology, diagnosis, and treatment of the disease, where the trainees can sit down and teachers can take advantage of the Microsoft Office PowerPoint (PPT) slide or multimedia video to explain the procedure or through problem-based learning (PBL) discussion pattern about the trainees' own concerns [7]. After theoretical teaching, the residents perform practical training for hysteroscopy surgery.

Recently, there is a common opinion that teaching is also an essential part of residents' responsibility in general surgery [8]. There are residency programs abroad called resident-as-teacher programs (RATPs) to equip residents with required teaching skills [9, 10]. However, RATPs in gynecologic residencies are not well characterized [11]. Most medical students who enter residency received no formal training regarding teaching [12]. On the other hand, although the importance of resident physician training has been well demonstrated, only a few formal hysteroscopic training opportunities exist for residents to learn effectively, which means that the operator should be familiar with assembling the elements of the operative and the power sources as well as technical operation skills [5]. Residents did clinical rotation one after another in different wards during different periods; they might not have attended all the topics of hysteroscopy TRs before they actually operate. Teaching quality assurance during hysteroscopic endoscopy often necessitates guidance from an expert instructor to explain the surgical procedure and considerations to the residents' side by side [13]. The main educational components include the anatomy of the female pelvis, pathophysiology of disease and diagnoses, operative indications, contraindications, limitations, and possible complications of every HS, together with full knowledge of the prevention, early recognition, and management of complications [5]. In this project, we planned to arrange an HS training project for residents including TR managed by senior residents themselves as well as tutor guided HS operation and discuss if this pattern of TR is more important for practical training in hysteroscopy surgery for obstetrics and gynecology resident physicians. Our aim was to assess the effectiveness of teaching techniques for educators not only to teach residents how to accomplish an operation but also to ensure that they acquire theoretical and basic clinical knowledge in a hysteroscopy teaching program.

## 2. Methods

### 2.1. Residents and Teachers Attending the Program

We invited 4 attending gynecologic surgeons as directors and 12 residents, including 7 junior residents (J1, J2, J3, J4, J5, J6, and J7) and 5 senior residents (S1, S2, S3, S4, and S5) to participate in this study. J1–J4 were in Grade 1, and J5–J7 were in Grade 2, while all the senior residents S1–S5 were in Grade 3. The residents did hysteroscopic operation during their clinical rotation in the gynecological ward and attended all of the TR courses even if they were in other wards. The gynecologic surgeons who attended the SRT program participated as educators, among whom 2 were males and 2 were females.

### 2.2. Teaching Round (TR)

All resident physicians had attended TRs through which theoretical knowledge, operational procedures, and precautions were taught. Typical hysteroscopy cases among hospitalized patients were selected under the guidance of a responsible senior physician, and all the 6 courses of TR were directed by the 5 senior residents and divided into the following three sections within 1 hour. Firstly, in the classroom, the leader explained the purpose of the rounds and the key knowledge that the students need to grasp for 10 min. Then, the residents reported the medical history and performed physical examination at the bedside for 15 min [14]. Finally, the leader guided the residents to conduct systematic and detailed summaries and discussions through either PPT descriptions, multimedia video, or PBL discussion for 35 min (Table 1). After that, we carried out theoretical tests within one week after each TR to observe the mastery degree of the basic theoretical and operational knowledge of hysteroscopy among students. Scores were according to the mastery degree of the knowledge: Very Good: 18–20; Good: 15–17; General: 10–15; Bad: <10 (Table 2).

### 2.3. Hysteroscopic Operation

To prepare for the practical hysteroscopic operation, the resident surgeons were instructed to review the patient's history, diagnosis, contraindications, and indications of HS. Over a period of 12 months, 112 patients met the inclusion criteria. Sixty-two patients were operated by 7 junior residents and fifty patients were operated by 5 senior residents. All HS procedures were performed by residents under the supervision of general gynecologists who were arranged to teach on a one-on-one basis. Our study was approved by the Ethical Committee on Human Research of Shanghai General Hospital affiliated to Shanghai Jiao Tong University.

### 2.4. Survey on the HS Teaching Program

We gave evaluation based on the mastery degree of the students after the HS teaching program. Quality of teaching was assessed by the mastery level of residents in HS using the analytic hierarchy process (AHP) methodology (Table 3) [15]. We also generated open-ended questions based on TR and HS to develop the question route. The survey also included questions about attitudes toward teaching by residents and teaching climate. The answers were collected and analyzed regarding the opinions between two groups of junior and senior residents as well as the teachers (Table 4).

### 2.5. Statistical Methods

All enrolled attending resident physicians and doctors abstracted the medical records. Comparisons between groups of junior and senior residents in the patient age, year of menopause, whole operation time, the volume of distension fluid used in the HS operation, and the intrauterine depth were performed by the *t*-test. Uterus position, postoperative vaginal bleeding, and pathology were performed by the Mann–Whitney test, and skewed data were compared using Fisher's exact test. All *p* values less than 0.05 were considered statistically significant, and all *p* values were two-sided.

## 3. Results

### 3.1. Resident-as-Teacher Model Teaching Rounds (TR)

There is, in particular, unique challenges for trainees to follow a clear educational plan in advance in order to gain specific theoretical knowledge prior to performing a hysteroscopic surgical intervention through TR. In general, we talk about theoretical knowledge in TR which was taught by the teachers before the surgery. Different from the traditional mode, in this program, a 2-month teaching rounds (TR) program was managed by senior residents though the PBL pattern. Senior residents have attended similar TR courses when they were in their junior grade given by the teachers; thus, this time, it was their turn to act as leaders to hold the course. Each teaching round had a focus on meeting the demands of different development strategies for trainees so that they learn effectively. Residents analyzed the causes, symptoms, signs, ultrasound reports, lab data, diagnosis, and treatments that require further observation, which could help in hospitalization at the bedside (Table 1). The teacher's role is to guide the students learning at the bedside. All the TR utilized PBL discussions were based on specific cases and responses from the focus group discussions, the students conducted discussions on points such as “Is it suitable for HS?” and “What are the complications of HS?”, and finally, the group leader reviewed the analysis. During the PBL class, the leader combined theoretical knowledge with practical operations to stimulate and attract students' enthusiasm as well as to improve the residents' abilities to handle practical problems. The teachers also called tutors engaged in discussions to make students' discussion on the main topic.

Observation of the actual operation could be a powerful tool to develop teaching skills, and the standardization process consisted of a videotaped HS by an experienced teacher so that the trainees could follow the procedure. Video-based education has potential use in surgical education as trainees face significant barriers in their practice. The use of video settings offered sufficient opportunities to integrate theoretical and clinical understanding. Hysteroscopic two-dimensional image display system videos were commonly used for residents who were unfamiliar with hysteroscopy.

Within one week after each TR, we arranged a test to observe if residents have grasped the content of the lesson and to evaluate the effect by different speakers. The teachers explained and reviewed the answers. As to junior residents, they got the mean score of 10.7 in the first test and 14.4 in the second test, and we observed that the more courses the residents attended, the higher scores they achieved among junior residents (Table 2). Senior residents received higher scores (mean, 104) than junior residents (mean, 92).

### 3.2. Hysteroscopic Surgery (HS)

After the students had mastered theoretical knowledge related to hysteroscopy through TR, they tried to engage in the hysteroscopy practical training stage. We arranged suitable HS cases for residents to operate according to the European Gynecologic Endoscopy Association. The training in hysteroscopy surgery is divided into the following three levels: primary level, which is suitable for the junior and senior residents, mainly for the diagnosis and simple operation, such as intrauterine fixed-point biopsy, removal of an intrauterine device, and mild intrauterine adhesions [16]. In order to maintain operational skills, all the residents completed the whole operation with the tutor accompany beside, guiding and solving problems at any time during the surgery.

Educators were encouraged to teach trainees hands-on [17], cultivate good operating habits in the residents, and correct the wrong methods in time. For example, the junior learner J1 and J2 in grade 1 was taught to explore the uterine cavity or to dilate the cervix “hand by hand” under the guidance of a directing teacher for the first time. Teachers helped to develop the residents' control and direction of the hysteroscopic operating device in the uterine cavity. When the residents faced difficultly in the removal of intrauterine device and extraction of endometrial polyps, the teachers would help them to locate with a clamp.

The directors try to avoid the occurrence of intraoperative complications, including controlling the surgery time to less than 1 h to minimize the occurrence of complications. In clinical observation, fluid overload caused by longer operation time means excessive use of fluid in patients in HS which usually results in the symptom of bloating as a result of the fact that the fluid in the uterine cavity enters the pelvis through the fallopian tube. In addition, in order to avoid serious complications following hysteroscopy such as air embolism or cutting excessive myometrial tissue, we should remove the air in the tube and control the pressure of the expansion valve and the flow rate of the perfusate as well as the cutting depth.

**Figure 1:**
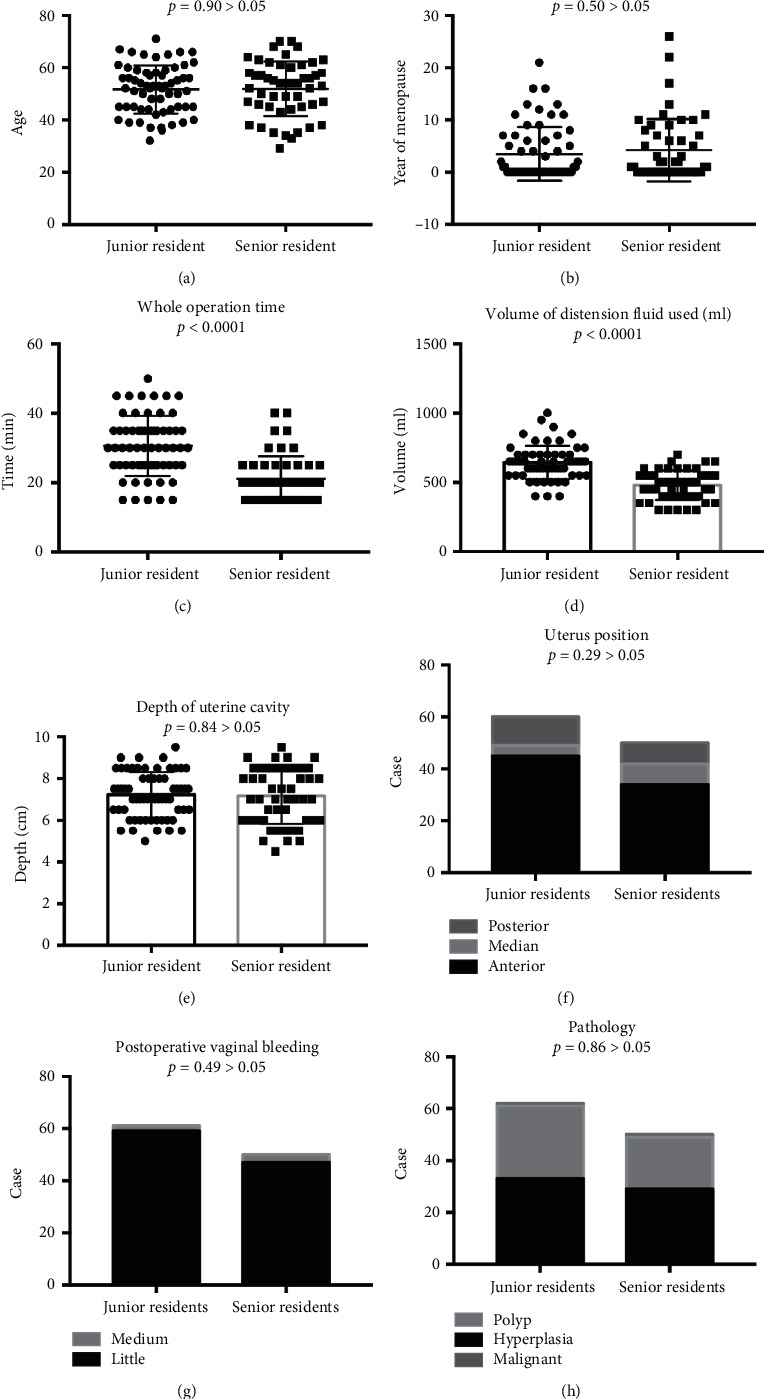
Comparison of patient age (a), menopause age (b), whole operation time (c), distension fluid (ml) (d), intrauterine depth (e), uterus position (f), postoperative vaginal bleeding (g), and pathology (h) between the two groups.

In order to explore the efficiency of tutor guided HS, after the operation was completed, procedure notes were reviewed with respect to trainee participation, and postoperative analysis was performed. The mean patient age was 51.78 years (range = 29–71 years), the mean menopause age was 3.8 years (range = 0–26 years), and the mean intrauterine depth was 7.22 cm (range = 5–9.5 cm). In this patient population, 62 procedures (60.6%) were performed by involving junior resident trainees, and 50 procedures (39.4%) were performed by involving senior resident trainees. Patient characteristics were summarized in Figure 1. There was no significant difference between junior residents and senior residents in terms of patient age (51.68 ± 1.14 versus 51.92 ± 1.38, *p*=0.90) (Figure 1(a)) and menopause age (3.48 ± 0.65 versus 4.20 ± 0.80, *p*=0.50) (Figure 1(b)). Since the directing teacher guided the residents to conduct the operation on-side, we compared operative time and distension fluid to calculate the time teacher spent to direct them besides. There were statistically significant differences in the whole operation time (min) and volume of distension fluid used (ml) between junior residents and senior residents. The whole operation time with the involvement of senior trainees was more likely to be less than that of junior trainees (30.56 ± 1.08 min versus 21.00 ± 0.92 min, respectively, *p* < 0.05) (Figure 1(c)). Junior residents involved in the procedures were more likely to use a greater volume of distension fluid (ml) than senior residents when conducting HS procedures (642.74 ± 14.82 ml versus 480.00 ± 14.76 ml, respectively, *p* < 0.05) (Figure 1(d)), mainly because the tutor spent more time to instruct junior residents than senior residents. In addition, we did not find any correlation of procedures performed with the involvement of junior residents and senior residents with intrauterine depth (7.22 ± 0.13 versus 7.17 ± 0.18, *p*=0.84) (Figure 1(e)), uterus position (*p*=0.29) (Figure 1(f)), postoperative vaginal bleeding (*p*=0.49) (Figure 1(g)), and pathology (*p*=0.86) (Figure 1(h)). Only one case of uterine perforation occurred after HS conducted by a senior resident to transit hysteroscopy surgery in all operations (0.89%) (Supplemental File 1).

### 3.3. Survey and Feedback of the HS Teaching Program

The time of the TR courses when each resident participated in the gynecological ward was shown in Table 3. The quality of teaching was predominantly assessed; the teachers gave the evaluation based on the mastery degree of the students, which was divided into unskilled, general, and good according to the residents' control and direction of the hysteroscopic operating device in the uterine cavity, the ability to conduct uterine curettage, and the whole operation time used. We observed that residents who participated in more TR before they finished their HS program conducted more skilled hysteroscopic operation (Table 3). As to the junior residents J1 and J2, they were unskilled at the initial time to do uterine curettage; however, with the increase of time of attending TR, junior residents J3, J4, J5, J6, and J7 became more and more skilled. Senior residents had attended similar TR when they were junior, so most of them were skilled (Table 3).

When the HS program finished, we ranked a list of potential concerns through a questionnaire focusing on different attitudes and effects between TRs managed by the teachers and residents which concluded challenges to good clinical rounds and HS based on responses from the focus group discussions (Table 4). All the residents believed that TR could help improve their surgical skills in HS. Junior residents preferred video-based TR because they could follow the process or review the videotapes of complex surgical procedures. The senior residents largely felt more comfortable with their role as surgeons or teachers through the PBL model, since they well prepared the basic knowledge and discussed their interesting content, and they were more skilled in conducting the hysteroscopic operation. Both teachers and students formed a good motivation surrounding during teaching time. Most junior residents hoped to finish all the TR courses before they operate, while senior residents consider that it was difficult to finish all the lessons before they operate because of the actual rotation plan. All the residents believed the HS teaching program was focused on their own concerns. As for actual operation, junior residents regarded that one of the most difficult parts of hysteroscopy was dilation of the cervix; therefore, the instructors would help them adjust the direction when they had difficulty in clamping the cervix and exploring the uterine. Another difficult thing for residents was the removal of intrauterine device and extraction of endometrial polyps because they usually felt hard to locate with a clamp (Table 4). All the senior residents felt that teaching was an important part of the senior resident's job.

## 4. Discussion

In the clinical teaching process, we were usually confused with the situation that although the teacher teaches the same contents, different students had different comprehension and master levels. Was this just because students had different degrees of ability or it also related to teaching methods? We thought it was the latter. The Accreditation Council for Graduate Medical Education (ACGME) (http://www.acgme.org) is responsible for the management of training residents to acquire advanced skills in the aspect of patient care, medical knowledge, evidence-based practice, communication skills, and system-based practice [18]. Generally, gynecologic SRT units in teaching hospitals use traditional teaching methods and encourage young doctors to develop clinical skills and participate in surgery which is classified into the following three categories: (1) teaching: the verbal explanatory stage like TRs, (2) directing: the verbal commanding stage, and (3) assisting: encompassing the physical guidance stage [19]. In our HS program, the teachers acted as tutors firstly to guide the residents analyzing the causes, symptoms, signs, ultrasound reports, lab data, diagnosis, and treatments in TR; next, tutors teach the trainees HS principles and “hand by hand” skills like instructing the residents' control and direction of the hysteroscopic operating device in the uterine cavity in the operating room, resulting in the fact that senior resident trainees were more skillful with the involvement of HS.

The use of clinical rounds, as an integral part of clinical teaching to help residents acquire essential skills of practicing medicine, is critically important. The father of modern medicine, William Osler, highly acknowledged bedside teaching in modern medicine: “To study the phenomena of disease without books is to sail an uncharted sea, while to study without patients is not to go to sea at all.” It was noted that “Twelve Tips to Improve Bedside Teaching,” which included preparation, priming of the patients, assignment of roles, the establishment of expectations, roadmap, the focus of the encounter, patients' notes, bedside teaching, role model, summary, feedback, and reflection, were conducive for residents skilled in basic operations [20]. Different forms of clinical rounds help residents identify the key areas in history taking and physical examination techniques; thus, the residents were familiar with data gathering and clinical decision making [21]. The teachers' role is to guide the students' learning at the bedside; they not only foster the residents' ability to perform physical examination skills but also help them to gain more experience.

Furthermore, teaching by residents has demonstrable value since the students' affective domain cannot be ignored [22]. Residents' learning was not only dependent on the teaching expertise of their attending surgeons but also more likely to be effective if teaching focused on their own concerns [23] and fulfill resident-as-teacher role [24, 25]. The American College of Surgeons has implemented a highly rated and well-established annual 3-day program at their Chicago headquarters to help prepare surgical residents to be more effective leaders and educators [26]. Also, the ACGME requires senior residents to complete a minimum of 25 surgical cases in which they act as a teaching assistant [27]. We found that directors in China recognized the value of resident teaching, but most did not have established formal curricula to help residents become effective educators. Reasons might include the resident's lack of teaching confidence or motivation to teach in the absence of formal teaching training [28, 29]. Therefore, our program invited senior residents to take charge of the TR in order to motivate them explicitly, since as they prepared the lecture, they spent more time on checking the reference and textbook. In the test, senior residents received higher scores (mean, 104) than junior residents (mean, 92). Senior residents may have implemented the similar TR course when they were in their junior grade, so in our HS teaching program, they dominated the lecture and showed an increased understanding of TR and operation for the following topics: (1) knowledge gaps related to education, (2) the role of education among academic surgeons, (3) educational tools to improve teaching performance, and (4) perceived knowledge and attitudes toward teaching in the operating room. In addition, the PBL discussion method has played specific roles to maintain the interest of each resident, changing the learning process from accepting basic theoretical courses to obtain cognitive, associative, and autonomous skills. PBL also provided a good effect on clinical teaching in our program and broke down barriers between educators and learners, which started with clinical practical problems and developed the theme with more detailed practical tips and up-to-date references. After TR, the observers gave the residents an exam to test if the residents had grasped the knowledge. We observed that the more courses the residents attended, the higher scores they achieved among junior residents; senior residents received higher scores than junior residents. Based on the elicited responses from our participants, all the residents believed that TR could help improve their surgical skills in HS, since during TR, expert surgeons were able to explain actual surgical cases as a deliberate part; thus, residents could gain a more comprehensive understanding of the indications and contraindications for HS as well as of prevention and management of complications. Therefore, we considered that teaching intervention is the reason for the improvement in the mastery level of surgery.

Checking rounds is an assessment tool in surgery [30]. Side by side teaching in the operation room is the time when most of clinical encounters and training between medical teachers and students occur. For beginners, there might be a lack of understanding about the strength and depth of the uterus during the operation; thus, in practice, the educators explained the principle and function of hysteroscopy instruments in the operating room. Also, videotaped, physical “moment-to-moment” teaching exchanges between the surgical attending physicians and their trainees could classify live surgical teaching behaviors [31]. Watching an instructional video before surgery may shorten the learning curve of trainees and improve safety [32, 33]. On the other side, teachers thought that completing the whole operation with explanation was important to achieve excellent surgical teaching behaviors, and longer operation time and higher volume of distension fluid (ml) were required by junior residents when they conducted hysteroscopic surgeries as teachers spent more time to teach them the technique. After that, residents are dominant most of the time in the surgery, and teachers are observers of practices so that the residents could have a good chance and take the responsibility for the operation.

However, there were limitations in our study including small sample size, thereby limiting the statistical difference between groups. Additional studies are recommended to assess the efficiency of hysteroscopy teaching under faculty supervision during the basic surgical procedure. The residents should also be able to perform HS efficiently when they operate independently in the future. Larger studies are needed to further explore the potential links among on-round TR, acquisition of high-value operational principles by the trainee, and patient outcomes, and more medical teachers should take part in faculty development programs.

## 5. Conclusion

Overall, surgical residents train and teach both in and outside of the operating room. It was noted that the higher the efficiency of teaching, the better the proficiency for surgery. It is important to improve the theoretical knowledge of resident physicians in the surgical department before the actual operation. This study acknowledges better characterization of the link between the use of TRs dominated by the residents themselves and tutor guided surgical skills. By adopting and promoting interprofessional collaborative practice model, the quality and effectiveness of bedside TR and HS guidance could be improved for the benefit of trainees and the instructor team as a whole.

## Figures and Tables

**Table 1:** Focus of the categories of teaching rounds.

Case	Date	Theme of TR	Diagnosis	Focus	Dominant	Tools
1	2018.8	Indications and contraindications of hysteroscopic surgery	Dysfunctional bleeding, endometrial thickening	Treatment and diagnostic value of hysteroscopy in endometrial diseases and submucosal fibroids; patients with the poor general condition are a contraindication for hysteroscopy	Resident, S1	PPT
Test 1: Treatment and diagnostic value of hysteroscopy in endometrial diseases?						

2	2018.10	Hysteroscopic surgical procedure and instrument assembling	Endometrial polyps	Hysteroscopic procedures: Disinfection, dilatation of the cervix, etc. After observing the location of endometrial polyps, clamp the endometrial polyps by an oval clamp	Resident, S2	Video, PPT
Test 2: Hysteroscopic procedures.						

3	2018.12	Hysteroscopic resection of uterine submucosal fibroids	Uterine submucosal fibroids	Cut the submucosal fibroids in the uterine cavity by an electrode ring	Residents S3	Video, PPT
PBL questions:						
(1) What is the indication and complication of using the electric-cutting function of hysteroscopic surgery?						
(2) How to prevent the complication of using the electric-cutting function of hysteroscopic surgery?						
(3) How to use the electric-cutting function of the hysteroscopic device?						
Test 3: Indication and complication of using the electric-cutting function of the hysteroscopic device.						

4	2019.2	Treatment of hysteroscopic complications	Postmenopausal endometrial thickening	Slowly dilate the cervix, explore the location of the uterus, etc. to prevent uterine perforation	Residents S4	PPT
Test 4: Treatment of hysteroscopic complications.						

5	2019.4	Problem-based learning (PBL) discuss	Endometrial cancer	Scratch gently to prevent uterine perforation, control the pressure of the uterine cavity to prevent tumor tissue from escaping through the fallopian tube	Residents S5	Video, PPT
PBL questions:						
(1) How to prevent uterine perforation?						
(2) If uterine perforation happened in hysteroscopic produce, how to solve?						
(3) How to communicate with patients and their families to explain the complications?						
Test 5: How to prevent uterine perforation?						

6	2019.6	The impact of hysteroscopy on the diagnosis of mediastinal uterus	Mediastinal uterus	Perform MRI examination in advance	Resident, S1	PPT
Homework:						
(1) What is the impact of hysteroscopy on the diagnosis of the mediastinal uterus?						
(2) How to resect mediastinal uterus?						
Test 6: The impact of hysteroscopy on the diagnosis of mediastinal uterus						

**Table 2:** Theoretical test scores within one week after each teaching round.

No	Grade	Sex	Test (score)						
Junior residents			1 (20)	2 (20)	3 (20)	4 (20)	5 (20)	6 (20)	Total

J1	1	Male	10	13	13	15	15	18	84
J2	1	Male	11	14	15	17	17	18	92
J3	1	Female	10	15	16	18	18	17	94
J4	1	Female	9	13	14	16	17	17	86
J5	2	Female	12	14	15	16	19	17	93
J6	2	Female	12	15	16	18	18	16	95
J7	2	Female	11	17	17	18	19	18	100
Mean score			10.7	14.4	15.1	16.9	17.6	17.3	92

Senior residents			1 (20)	2 (20)	3 (20)	4 (20)	5 (20)	6 (20)	Total
S1	3	Female	14	16	17	18	17	19	101
S2	3	Female	16	17	18	19	19	18	107
S3	3	Female	15	17	17	18	17	17	101
S4	3	Female	15	16	18	20	18	18	105
S5	3	Female	16	18	19	17	18	18	106
Mean score			15.2	16.8	17.8	18.4	17.8	18	104

**Table 3:** Mastery and the time of TR courses and operation cases when each resident participated in the gynecological ward.

No	Date of rotation in the gynecological ward	TR during HS program	Total HS case	Uterine curettage	Case of uterus disease and skilled level evaluated by the directed teacher						
					Level	Removal of the intrauterine device	Level	Endometrial polyps	Level	Mild intrauterine adhesions	Level
J1	2018.8–2018.10	2	7	4	Unskilled	1	Unskilled	2	General	0	
J2	2018.8–2018.10	2	8	3	Unskilled	2	General	3	General	0	
J3	2018.11–2019.2	4	8	5	General	1	General	2	Unskilled	0	
J4	2018.11–2019.2	4	6	4	General	0	General	2	General	0	
J5	2019.3–2019.6	6	10	4	Good	2	Good	3	General	1	Unskilled
J6	2019.3–2019.6	6	10	3	General	2	Good	4	General	1	Unskilled
J7	2019.5–2019.8	6	13	5	Good	2	General	5	General	1	General
		Total	62	28		10		21		3	

S1	2018.8–2019.2	4	9	4	Good	1	Good	4	Good	1	General
S2	2018.8–2019.2	4	11	4	Good	1	General	5	General	1	General
S3	2019.3–2019.8	6	9	5	Good	0	Good	4	General	0	
S4	2019.3–2019.8	6	10	6	Good	1	Good	3	Good	0	
S5	2019.3–2019.8	6	11	4	Good	1	Good	5	General	1	General
		Total	50	23		4		21		3	

**Table 4:** Different attitudes and effects between TRs managed by the teachers and residents.

No	Question to residents	Residents Answer	
		Junior residents	Senior residents

1	Do you think this kind of TR managed by the residents would help you improve your theoretical and practical knowledge in HS?	Yes	Help a lot, reviews of all details of HS were a valuable teaching experience, and we were motivated in discussion
2	Was the HS teaching program focused on your own concerns?	Yes, we were engaged in discussions, talking about basic theoretical and operational knowledge of hysteroscopy that attracted us	Yes, we also wanted to talk more about the key skills of HS
3	Do you hope to finish all the HS TR courses before you operate?	We hope to participate in more TR courses before we operate HS	Due to the actual rotation plan, it was difficult to finish all the HS TR course before we operate HS
4	What do you think is the most difficult part of hysteroscopy?	Dilation of the cervix, removal of intrauterine device, and extraction of endometrial polyps	Extraction of endometrial polyps and separation of intrauterine adhesions
5	Continued with question 4, how did your teacher help you solve this problem?	Adjusting the direction when tracking the cervix and exploring the uterine	Teach us how to use the clamp
6	Do you think that surgical skills were related to the guidance of the instructor?	Yes	Yes
7	When do you feel you were more familiar with the HS procedure in addition to clinical skills?	After learning the basic theoretical and operational knowledge of hysteroscopy through TR	Communicate with an experienced clinician and learn patient-management

No	Question to teachers	Teacher answer	

1	What are your experiences in guiding the residents related to bedside and operation teaching?	Focus the objectives of the TR and tried to have enough discussions which would help with transferability of the study	
2	Are there any ways to overcome the challenges in TR?	Guide senior residents well dominated TR through hands-on experiences	
3	What are the obstacles embedded in clinical education when teaching students on rounds?	Professionalism and commitment in gaining medical knowledge, and embedded in the affective domain of learning	
4	What are the skills of surgery teaching?	Complete the whole operation by explaining details about HS production	

## Data Availability

The datasets used and analyzed during the current study are available in the Supplementary Information files.

## Supplementary Materials

Supplementary Materials The datasets used and analyzed during the current study are available in the Supplementary Information files.
